# Preoperative Evaluation in Patients With End-Stage Renal Disease and Chronic Kidney Disease

**DOI:** 10.1177/1178632917713020

**Published:** 2017-06-28

**Authors:** Rabih Nasr, Sridhar Chilimuri

**Affiliations:** Department of Medicine, Bronx-Lebanon Hospital Center, Bronx, NY, USA

**Keywords:** ESRD, dialysis, preoperative, hypertension

## Abstract

Optimal preoperative management of dialysis patients remains challenging. Patients with end-stage renal disease (ESRD) have higher mortality in the perioperative setting compared with non-ESRD patients. However, it is well established that dialysis should be done on the day before surgery. Additional dialysis session prior to surgery does not improve outcomes. All dialysis patients should undergo blood work to check electrolytes and especially serum potassium prior to any surgery. Some medications, including angiotensin-converting enzyme inhibitors, angiotensin receptor blockers, and diuretics, should be stopped prior to surgery to minimize hemodynamic changes during surgery. The dialysis access should be carefully examined for any signs of infection. The arteriovenous fistula or graft should be evaluated for patency. Glycemic control in diabetic ESRD and chronic kidney disease patients is very important, and clinicians should be aware of the risk of bleeding and the appropriate analgesics that can be used in dialysis patients in the perioperative setting. In conclusion, preoperative evaluation in patients with ESRD should be a multidisciplinary approach.

## Introduction

There exist limited published data regarding the optimal preoperative management of dialysis patients undergoing surgery. In this review article, we will be discussing different aspects of preoperative management in a specific population, including patients with end-stage renal disease (ESRD) undergoing hemodialysis 3 times per week. The first section will be about mortality and morbidity, preservation of residual renal function, and the best timing of dialysis. The second section will discuss the preoperative evaluation, including history and physical examination of the dialysis access, blood work needed, complications such as bleeding, and drug dosing in patients with ESRD undergoing maintenance dialysis 3 times per week.

## Mortality and Morbidity

There is higher perioperative mortality in the ESRD population compared with the non-ESRD population.^1–3^ Deutsch et al^4^ demonstrated that dialysis patients have increased morbidity with increased pressor requirements, longer time on mechanical ventilation, longer intensive care unit (ICU) stay, and longer hospital stays when compared with patients with normal kidney function. There are numerous causes for the increased mortality and morbidity.

Some of the factors include the following:

- Dialysis and chronic kidney disease (CKD) patients have more fluid and electrolyte disturbances, which can occur at a higher rate in the perioperative period, especially hyperkalemia.^5^
- There is increased incidence of myocardial dysfunctions and coronary artery disease.
- Patients with ESRD and CKD have worsening bleeding complications, mainly due to platelet dysfunction.
- There is inadequate blood pressure control, which could be either hypotension or hypertension. This could occur intraoperatively and postoperatively, contributed by the pain and catecholamine surge after surgery.

## Best Timing of Dialysis Prior to Surgery

If a patient undergoes maintenance dialysis on Tuesday, Thursday, Saturday schedule, surgery should not be scheduled on Monday. Preferable timing of dialysis would be on the day before surgery.^6^ Additional dialysis session prior to surgery does not improve outcomes. If the surgery is emergent and dialysis needs to be done on the same day of surgery, then heparin should not be administered during dialysis. The prescription for dialysis is usually the same. Laboratory values, including serum calcium, potassium, serum urea nitrogen, creatinine, magnesium, bicarbonate, and phosphorus, should be carefully monitored and adjusted to use the appropriate dialysate calcium, potassium, and bicarbonate so that the patient would go to the operating room with near-normal plasma concentrations.

Regarding ultrafiltration, it should be adjusted to make sure the patient is close to dry weight before surgery.

In patients undergoing peritoneal dialysis, some experts increase the amount of dialysis 1 week before surgery. In regard to this approach, there exist no published data to support this. Some nephrologists do not increase the dialysis time.

In general, dialysis patients should be adequately dialyzed, be euvolemic, and have near-normal electrolyte panel before undergoing surgery.

## Preserving Residual Renal Function Prior to Surgery

Patients new to dialysis have residual renal function, which is extremely important for solute clearance and fluid balance, especially in the first 6 to 12 months after initiating dialysis. As previously demonstrated, residual renal function is associated with increased survival.^7^ Regarding the use of angiotensin-converting enzyme (ACE) inhibitors and angiotensin receptor blockers (ARB) prior to surgery, data remain conflicting. However, as it is well known that hemodynamic changes can occur during surgery, contributed by the above agents and the risk of hyperkalemia, stopping these agents before surgery is not uncommon and seems reasonable. Similarly, for diuretics, as they can induce electrolyte changes and hemodynamic changes, it is reasonable to stop them.

Hemodynamic instability will be reduced when these agents are stopped prior to surgery. With this approach, it is intended to minimize hemodynamic changes, which can occur intraoperatively and thus lead to further complications.

Stable patients after surgery can resume diuretics, ACE inhibitors, and/or ARBs.

## Preoperative Evaluation

All dialysis patients should have a history and physical examination; baseline electrolyte values, including serum calcium, phosphorus, magnesium, albumin, and glucose; and coagulation profile prior to surgery. It is crucial to monitor the phosphorus level before and after surgery as it may decrease postoperatively due to inadequate oral intake. Phosphate binders should be discontinued as they can result in lower phosphorus levels. In patients with diabetes mellitus, serum glucose should be carefully monitored before, during, and after surgery.

The dialysis access should be carefully examined for any signs of infection. The arteriovenous fistula or graft should be evaluated for patency.

Regarding the anemia status, if the hemoglobin is below the target in patients with ESRD, then iron studies should be examined and erythropoietin-stimulating agents can be given preoperatively.

There are 2 major indications for urgent preoperative dialysis, which are hyperkalemia and volume overload. No guidelines exist at present to set a maximum safe level of potassium before anesthesia induction.

Dialysis patients commonly have elevated blood pressure, which might require treatment prior to surgery. Initially, treatment of hypertension is directed toward optimizing volume status with effective ultrafiltration because most of the time volume overload is the most common cause of hypertension.

Therapy for hypertension might be necessary if the blood pressure remains elevated despite achieving optimal dry weight. Common intravenous agents that can be used for hypertension treatment include intravenous enalaprilat, labetalol, hydralazine (used concurrently with β-blockers), diltiazem, and/or nitroglycerin. If the patient is in the ICU, then intravenous nitroprusside or nicardipine can be used.

Transdermal clonidine would not achieve blood pressure control immediately and could be used postoperatively. However, long-acting antihypertensives should be avoided in the perioperative setting as they can cause hemodynamic instability and a higher risk of intraoperative hypotension. In general, clonidine and β-blockers should not be initiated preoperatively, but if the patients were already on them, then we should continue to avoid withdrawal symptoms.

Dialysis patients have increased risk of ischemic heart disease. Cardiovascular disease was thought to exist in 50% of dialysis patients undergoing surgery.^5,8^ There is no well-defined optimal preoperative cardiac assessment for dialysis patients, but it generally depends on the level of risk and requires risk stratification. Coronary artery disease and myocardial dysfunction result in significant morbidity and mortality in patients with ESRD. Cardiovascular disease remains the main cause of death in patients with ESRD.^8,9,10^ Not to forget to mention that dialysis patients tend to have chronic inflammation and malnutrition, which has been named as malnutrition-inflammation complex syndrome. This leads to further increased risk of cardiovascular mortality despite low body mass index, low serum cholesterol levels, and hypoalbuminemia.^11^ So it is crucial to realize that dialysis patients have increased cardiovascular risk in the perioperative setting although in the absence of traditional cardiovascular risk factors.

β-blockers should not be initiated before surgery, if patients are not already on them. Those who are already taking β-blockers should remain on them to prevent withdrawal. β-blockers can be used for rate control especially in the postoperative setting, where pain and catecholamine surge contribute to hypertension and hemodynamic instability.

Dialysis patients have an increased tendency to bleed.^12–15^ However, bleeding time is not recommended as a preoperative screening test. A normal bleeding time does not exclude prolonged bleeding complication during or after surgery. Multiple factors contribute to increased tendency to bleed, including platelet dysfunction. Some of the contributing factors for platelet dysfunction include the following: aspirin use, uremic toxin retention due to inadequate dialysis, anemia, and elevated parathyroid hormone. To limit uremic bleeding, some steps could be undertaken, which include increasing the hematocrit level by blood transfusion to target hemoglobin of 10.^16^ Desmopressin can be given intravenously or subcutaneously, cryoprecipitate may also be given, and, finally, dialysis is done to correct uremic platelet dysfunction. Regarding heparin, doses can be reduced by use of saline flushes during the hemodialysis treatment. Heparin with dialysis should be avoided 24 to 48 hours after major surgery. Discussion with the surgeon is very important.

A big proportion of patients with ESRD have diabetes mellitus. Glycemic control is crucial in the perioperative period. Some important points to consider in dialysis patients with diabetes mellitus are that they tend to be brittle, especially patients with type 1 diabetes mellitus. Important consideration not to overlook is that oral hypoglycemic agents have prolonged half-life in patients with ESRD and CKD, which could cause hypoglycemia. Consultation with diabetes specialist is advised.

In regard to intravenous access, it is recommended to use small caliber IV catheters. Internal jugular venous catheters should be placed if peripheral access is not available. Placing catheters in subclavian vein should be avoided at all times due to the risk of central stenosis. Central lines should not be inserted on the same side as arteriovenous access. Before going to the surgery, anesthesiologist should be aware of the patient’s vascular anatomy to help establish IV access and to minimize complications. It is always important to display a sign about the patient’s access side and to forbid blood draws and blood pressure measurement on the same side of access. Education of patients is crucial to always remind the health care professional not to use the arm with the access. Dialysis patients should not have peripherally inserted central catheter lines inserted unless they have short life expectancy, to preserve the veins for future arteriovenous access.

## Dosing of Drugs in Patients With ESRD

Patients with impaired kidney function have decreased renal excretion of drugs. Thus, the pharmacokinetics of medications is altered along with the metabolism, plasma protein binding, and volume of distribution.^17,18^ It is important to consider the metabolic pathway of depolarizing agents and analgesics in the perioperative setting.

There are 2 opioids which are of particular concern, including morphine and meperidine. Their metabolite accumulates in patients with CKD and ESRD and could lead to complications. Patients could be exposed to seizure if meperidine was used, as the metabolite normeperidine is a seizure-inducing substance. However, when morphine is used, its metabolite morphine-6-glucuronide is a highly active metabolite which could accumulate and lead to prolonged sedation.^19–21^ Therefore, morphine and meperidine should be avoided. The preferred analgesics are mainly fentanyl^22^ and hydromorphone.

Other available analgesics include nonsteroidal anti-inflammatory agents (NSAIDs). These agents could be used in patients with ESRD, but clinicians should be aware of increased gastrointestinal bleeding, especially in patients with ESRD, which limits their use. In patients with CKD, NSAIDs should be avoided due to increased renal toxicity causing acute kidney injury. Acetaminophen can be used without change in dosing.^23^ Tramadol can also be used in patients with ESRD.

Neuromuscular-blocking agents have altered metabolism and prolonged half-life in renal failure patients. It could be due to either decreased renal excretion or impaired active enzyme degradation. The depolarizing agents of choice include atracurium and cisatracurium. These are cleared by ester hydrolysis and are not affected by renal failure.

## Conclusions

Preoperative evaluation in patients with ESRD should be a multidisciplinary approach. However, it remains challenging and requires due diligence. Cardiovascular disease remains the most common cause of death in patients with ESRD, and careful risk stratification should be undertaken before surgery, even in the absence of traditional risk factors. Hyperkalemia and volume overload should be addressed before surgery and usually are corrected with dialysis. Glycemic control in diabetic ESRD and CKD patients is very important, and clinicians should be aware of the risk of bleeding and the appropriate analgesics that can be used in dialysis patients in the perioperative setting.
